# Mitotane-Induced Endocrine Alterations in Children with Adrenocortical Carcinoma: Clinical Implications from a 20-Year Retrospective Study

**DOI:** 10.3390/children12081031

**Published:** 2025-08-05

**Authors:** Gerdi Tuli, Jessica Munarin, Stefano Gabriele Vallero, Matilde Piglione, Eleonora Biasin, Luisa De Sanctis, Franca Fagioli

**Affiliations:** 1 Department of Pediatric Endocrinology, Regina Margherita Children’s Hospital, 10126 Turin, Italy; jessica.munarin@unito.it (J.M.); luisa.desanctis@unito.it (L.D.S.); 2 Department of Pediatrics, University of Turin, 10124 Torino, Italy; stefano.vallero@unito.it (S.G.V.); matilde.piglione@unito.it (M.P.); eleonora.biasin@unito.it (E.B.); franca.fagioli@unito.it (F.F.); 3 Department of Pediatric Oncoematology, Regina Margherita Children’s Hospital, AOU Città della Salute e della Scienza, 10126 Turin, Italy

**Keywords:** pediatric adrenocortical carcinoma, mitotane, endocrine effects

## Abstract

**Highlights:**

**What are the main findings?**

- Mitotane treatment is associated with high incidence of endocrine complications, in pediatric patients affected by adrenal carcinoma
- Serum mitotane levels show a positive correlation with the onset of precocious puberty and a negative correlation with central hypothyroidism

**What is the implication of the main finding?**

- Careful endocrine monitoring and a multidisciplinary approach occur to care throughout mitotane therapy in pediatric patients
- Strict monitoring of mitotane serum level is mandatory to avoid adverse effects

**Abstract:**

Background/Objectives: Mitotane is a key component in the treatment of adrenocortical carcinoma (ACC), but its endocrine side effects in children remain under-characterized. Methods: We conducted a retrospective analysis of 11 pediatric patients (6 males, 5 females) diagnosed with ACC and followed between 2000 and 2025. Seven received mitotane therapy. Data included age at diagnosis, treatment duration and dosage, serum mitotane levels, and endocrine complications. Results: The mean age at diagnosis was 6.6 ± 1.45 years, with a mean follow-up of 10.05 ± 2.45 years. Patients received mitotane for an average of 2.5 ± 0.54 years, with a mean daily dose of 2805.5 ± 145.82 mg and a mean serum level of 16.1 ± 5.92 mg/mL. All mitotane-treated patients developed adrenal insufficiency, requiring supraphysiological hydrocortisone replacement. Four also required mineralocorticoid therapy. Five developed precocious puberty; two males presented with prepubertal gynecomastia; three females were managed with GnRH analogs or aromatase inhibitors followed by estrogen receptor antagonists. Four patients developed central hypothyroidism, treated with levothyroxine. A positive correlation was found between mean serum mitotane levels and the onset of precocious puberty (*p* = 0.04), while mitotane levels correlated negatively with the development of central hypothyroidism (*p* = 0.001). Conclusions: Mitotane therapy in pediatric ACC is strongly associated with significant endocrine dysfunction. These findings emphasize the need for proactive, multidisciplinary endocrine management throughout treatment.

## 1. Introduction

Mitotane (o,p’-DDD) is an adrenolytic agent used primarily in the treatment of adrenocortical carcinoma (ACC), a rare endocrine malignancy. ACC commonly presents with hormone hypersecretion, particularly at initial diagnosis and in advanced stages. Prognosis remains poor, especially in metastatic or unresectable cases. While extensively studied in adults, the use of mitotane in pediatric patients has been less documented due to the low incidence of ACC in children, estimated at 0.2–0.3 cases per million per year [1,2]. Pediatric ACC (pACC), however, often displays distinct clinical and molecular characteristics, including a higher prevalence of hormone-secreting tumors and germline mutations [3,4,5]. The most frequently observed molecular pathogenic variant in pACC is TP53 R337H, which has been predominantly identified in Southern Brazil. This founder mutation is estimated to occur approximately 15 times more frequently in Brazilian patients compared to non-Brazilian cases [5]. In addition to TP53 R337H, other hereditary cancer syndromes, such as Li–Fraumeni syndrome and Beckwith–Wiedemann syndrome, have also been implicated in the pathogenesis of pACC, highlighting the critical role of genetic susceptibility in disease development. These differences call for tailored therapeutic strategies, within which mitotane may play a pivotal role [6].

In pediatric settings, based on a recent international consensus statement, mitotane is primarily indicated for all patients with advanced adrenocortical carcinoma (ACC), defined as surgically unresectable or metastatic disease, as well as in cases of incomplete resection and/or intraoperative tumor spillage. Additionally, mitotane is recommended for patients with stage III or IV ACC, even after complete surgical resection, and as neoadjuvant therapy when complete resection is not feasible [7]. The rationale for mitotane use lies in its selective cytotoxicity to adrenal cortical cells, effectively targeting residual tumor cells and micrometastases. However, its therapeutic window is narrow, requiring serum levels to be maintained between 14 and 20 mg/L to achieve optimal efficacy, while minimizing toxicity. This is particularly challenging in children due to pharmacokinetic variability, differences in metabolism, compliance issues, especially in very young children, and limited dosing data. The most commonly accepted starting dose is 50 mg/kg/day or 1500 mg/m^2^/day, which can be gradually increased up to 4000 mg/m^2^/day. To maintain therapeutic levels, the daily dose should be divided into 2–3 administrations [7,8].

Adverse effects in pediatric patients mirror those seen in adults but can be more pronounced due to developmental considerations [9,10]. Among the various adverse effects of mitotane—including gastrointestinal and neuropsychological symptoms—endocrine side effects are particularly common. Mitotane-induced inhibition of the adrenal cortex often leads to adrenal insufficiency, frequently requiring glucocorticoids and mineralocorticoids replacement therapy [11,12]. The strong induction of CYP3A4 by mitotane results in an increased metabolic clearance of glucocorticoids, necessitating supraphysiological replacement doses—typically about twice the standard requirement. In adults, European guidelines recommend initiating glucocorticoid replacement therapy on the first day of mitotane treatment. However, in pediatric populations, no current consensus exists on the optimal timing for initiating replacement therapy or monitoring protocols. Mineralocorticoid replacement is less frequently required, suggesting that the zona glomerulosa of the adrenal cortex is less susceptible to mitotane’s adrenotoxic effects. Mitotane also exerts an estrogenic effect through a receptor α-dependent mechanism, potentially causing peripheral precocious puberty in females and gynecomastia in males. Additionally, mitotane increases hepatic synthesis of sex hormone-binding globulin (SHBG), which may result in hypogonadism related symptoms due to reduced free sex hormones levels. Central hypothyroidism may also occur during mitotane treatment [13], likely as a result of increased levels of thyroxine-binding globulin and enhanced deiodinase activity, which can alter peripheral thyroid hormone metabolism. Growth and bone health should be carefully monitored during treatment, as mitotane itself—together with associated pubertal disorders and the use of supraphysiological doses of glucocorticoids—may negatively impact skeletal development and linear growth [14].

Given the frequent and significant endocrine adverse effects associated with mitotane treatment in pACC, we conducted a retrospective case series analysis spanning a 20-year period.

## 2. Materials and Methods

We retrospectively collected demographic and clinical data from all patients diagnosed with histologically confirmed pACC who were treated with mitotane and followed at both the Department of Pediatric Oncohematology and the Department of Pediatric Endocrinology of the Regina Margherita Children’s Hospital in Turin. The study period spanned from 2005 to 2025. Inclusion criteria were age below 18 years at diagnosis, availability of complete clinical records, and treatment with mitotane as monotherapy or in combination with surgery and/or chemotherapy with a minimum follow-up duration of 6 months from mitotane initiation. Patients with incomplete records or who were lost to follow-up before treatment initiation were excluded. Data were collected from medical records using a standardized data extraction form.

The following variables were recorded:

- Demographics: Age, sex, weight, and genetic background (if available).
- Clinical presentation: Hormonal secretion profile, tumor size, staging at diagnosis according to the ENSAT classification.
- Treatment details:
  o Surgical intervention (yes/no, type, and margin status)
  o Chemotherapy protocols, if administered
  o Mitotane dosing, administration schedule, and treatment duration
- Monitoring: Plasma mitotane levels (measured at regular intervals), endocrine function, and adverse events.
- Outcomes: Treatment response (complete response, partial response, stable disease, or progression), event-free survival, overall survival, and treatment-related toxicity.

Mitotane was initiated in patients classified as stage III, IV, when complete resection was not feasible and in case of tumor spillage. Initial dose was 50 mg/kg/day or 1.5 g/m^2^/day, with dose adjustments based on clinical tolerance and therapeutic drug monitoring. Plasma mitotane levels were measured every 2–4 weeks using the Lysosafe monitoring system (www.lysosafe.com, accessed on 1 April 2025). The target plasma concentration was defined as 14–20 mg/L.

Endocrine assessment included measurement of luteinizing hormone (LH), follicle-stimulating hormone (FSH), oestradiol, testosterone, androstenedione, dehydroepiandrosterone sulfate (DHEAS), and prolactin (PRL) in cases of suspected precocious puberty. In addition, thyroid function was monitored monthly by assessing thyroid-stimulating hormone (TSH), free triiodothyronine (FT3), and free thyroxine (FT4). All hormone levels were measured using chemiluminescent immunoassay.

Glucocorticoid replacement therapy at twice the standard dose was initiated on the first day of mitotane treatment in all children without pre-existing cortisol excess. In patients presenting with hypercortisolism, adrenal function was monitored every 2–4 weeks by measuring morning serum cortisol and adrenocorticotropic hormone (ACTH) levels. Mineralocorticoid function was also evaluated every 2–4 weeks by measuring aldosteron, renin and electrolytes. Fludrocortisone replacement therapy was initiated when mineralocorticoid deficiency was confirmed.

Statistical analysis and graphs were performed by Graphpad 7 software (GraphPad Software, La Jolla, CA, USA) using the Pearson coefficient for correlation and Student’s t test for comparison of means. Significant difference among the means was considered if *p* < 0.05.

The study was conducted in accordance with the Declaration of Helsinki, and approved by the Ethics Committee of Città della Salute e della Scienza (protocol code 003857, 5 May 2024) Parental informed consent was obtained in all cases for the retrospective data collection and publication.

## 3. Results

Clinical data were collected from 11 patients (5 males, 6 females) with histologically confirmed pACC. Among these, seven patients received treatment with mitotane and were included in the present study. The mean age at diagnosis was 6.5 ± 1.45 years. According to the ENSAT staging system, four patients were classified as stage II, three as stage III, and four as stage IV. Hormonal evaluation revealed androgen excess in seven patients, while mixed cortisol and androgen secretion was observed in four patients (all classified as stage II). The mean follow-up duration was 10.05 ± 2.45 years.

Five patients received first-line chemotherapy with etoposide, doxorubicin, and cisplatin (EDP) regimen. One patient subsequently underwent second-line treatment with vincristine, followed by a third-line regimen comprising etoposide and carboplatin. Another patient received second-line therapy with gemcitabine and capecitabine, and third-line treatment with temozolomide succeeded by pembrolizumab; in this patient, local radiotherapy was also administered due to partial surgical resection of the neoplasm. Three patients did not receive additional systemic therapy beyond the initial EDP regimen.

All stage III (n = 3) and stage IV (n = 4) patients received mitotane therapy for an average of 2.5 ± 0.54 years. The mean daily mitotane dose was 2805.5 ± 145.82 mg with a mean serum level of 16.1 ± 5.92 mg/mL.

Endocrine adverse effects associated with mitotane treatment are summarized in Table 1. All patients received supraphysiological doses of hydrocortisone (36.5 ± 0.87 mg/m^2^) at the initiation of mitotane therapy. Mineralocorticoid replacement therapy was required in four patients (57.1%). Among the observed adverse effects, prepubertal gynecomastia occurred in two male patients, while peripheral precocious puberty (PPP) was identified in three female patients. No specific treatment was initiated for the two male patients presenting with gynecomastia. In one case, the disease was at a very advanced stage, resulting in a fatal outcome; in the other, the decision was based on the patient’s age at onset (13 years) and progress to full puberty was observed. Of the three females with PPP, two developed subsequent central precocious puberty and were treated with GnRH (LHRH) analogs, while the remaining patient, who exhibited only PPP, was initially treated with aromatase inhibitors followed by estrogen receptor antagonists. Central hypothyroidism was identified in four patients, all of whom were initiated on levothyroxine (L-thyroxine) replacement therapy.

A significant positive correlation was observed between mean serum mitotane levels and the occurrence of precocious puberty (*p* = 0.04), while a significant negative correlation was found between mean mitotane levels and the development of central hypothyroidism (*p* = 0.001).

## 4. Discussion

Mitotane remains a cornerstone in the adjuvant treatment of pACC, particularly in patients with advanced-stage disease [15]. However, its administration is frequently associated with significant endocrine adverse effects due to its adrenolytic properties and its complex impact on the hypothalamic–pituitary axis and peripheral endocrine functions [7].

While the endocrine effects of mitotane are well described in adults, data in children remain limited due to the rarity of pACC [16,17,18]. Although several authors have suggested a potential role of mitotane in the development of endocrine dysfunction, comprehensive case series or cohort studies addressing these issues in the pediatric population are scarce. To our knowledge, the largest previously reported case series included four female patients, all of whom presented with precocious puberty [16]. In that cohort, adrenal insufficiency occurred in all cases, and central hypothyroidism was reported in one patient.

In our cohort, all patients receiving mitotane were treated with supraphysiological doses of hydrocortisone, reflecting the drug’s potent suppression of endogenous cortisol production. Replacement therapy was initiated in all patients who did not exhibit hypercortisolism at diagnosis. Mineralocorticoid replacement was required in more than half of the patients, highlighting the frequent occurrence of primary adrenal insufficiency and the need for careful monitoring and appropriate hormonal replacement. Of the three patients who discontinued mitotane, two were able to stop replacement therapy for adrenal insufficiency after 6 months and 2 years, respectively. One patient remains on replacement treatment 4 years after mitotane withdrawal.

Precocious puberty was observed in five patients (71.4%). Notably, prepubertal gynecomastia developed in male patients, while female patients exhibited peripheral precocious puberty (PPP), consistent with prior reports describing mitotane-induced alterations of sex steroid metabolism. The treatment of gynecomastia in male patients remains controversial and is largely influenced by the age at onset. In very young children, medical therapy with aromatase inhibitors or selective estrogen receptor modulators may be considered to prevent progression to full pubertal development. However, in our cohort, neither of the two male patients presenting with gynecomastia received specific treatment. Furthermore, we did not identify any published data addressing the management of gynecomastia in prepubertal males with pediatric adrenocortical carcinoma. In two of the three female patients, PPP evolved into central precocious puberty, requiring treatment with GnRH analogs, which suggests a potential triggering role of peripheral sex hormone excess on hypothalamic activation. Another female patient with isolated PPP was initially treated with the aromatase inhibitor anastrozole. Six months later, progression of telarche and uterine length prompted initiation of the estrogen receptor antagonist fulvestrant. After six months, no further pubertal progression was observed. To date, this is the largest case series reporting precocious puberty during mitotane treatment and the first to describe the use of fulvestrant therapy for PPP during mitotane treatment.

Central hypothyroidism was observed in four patients (57.1%). Among the two patients who discontinued mitotane, one stopped LT4 treatment after 3 years, while the other remains on LT4 therapy 6 months after mitotane withdrawal.

A particularly relevant finding in our study was the positive correlation between mean mitotane serum levels and the onset of precocious puberty (*p* = 0.04), underscoring a possible dose-dependent effect of mitotane on gonadal steroid pathways. Conversely, we identified a negative correlation between mitotane levels and the development of central hypothyroidism (*p* = 0.001). Although the mechanism remains unclear and may be related to the limited dimension of the cohort, it is plausible that mitotane’s effect on pituitary function contributes to central hypothyroidism, necessitating levothyroxine replacement in affected patients.

Pediatric patients affected by pediatric adrenocortical carcinoma (pACC) may also receive chemotherapy regimens that include agents other than mitotane. These additional chemotherapeutic agents can interfere with the endocrine system—particularly the thyroid–hormone axis—potentially exposing patients to additional risk factors for developing endocrine disorders and general toxicity [19].

These findings emphasize the need for comprehensive endocrine surveillance during mitotane therapy in children, including regular assessments of adrenal, thyroid, and gonadal axes. An additional important effect of mitotane that warrants evaluation in the pediatric population is its potential impact on bone health. Although this parameter was not assessed in the present study, it represents a relevant area for future investigation.

The management of mitotane-induced endocrine complications requires a multidisciplinary approach, involving pediatric endocrinologists to ensure timely diagnosis and appropriate treatment, to improve quality of life and long-term outcomes in this vulnerable population.

Further research in larger cohorts is warranted to better elucidate the mechanisms underlying mitotane-induced endocrinopathies and to define optimal hormonal replacement strategies and monitoring protocols during treatment.

## 5. Conclusions

In pediatric patients with adrenal carcinoma, mitotane treatment is associated with a significant incidence of endocrine complications, particularly adrenal insufficiency, precocious puberty, and central hypothyroidism. Serum mitotane levels show a positive correlation with the onset of precocious puberty and a negative correlation with central hypothyroidism. These findings highlight the importance of careful endocrine monitoring and a multidisciplinary approach to care throughout mitotane therapy in pediatric patients.

## Figures and Tables

**Table 1:** Demographic and clinical data of pediatric patients treated with mitotane for adrenal adenocarcinoma.

Case	Age at Diagnosis	Onset Symptoms	Stage	Surgery	Treatment	AI	PP	CH	Genetics	Follow-Up (Years)	Outcome
1 (M)	3.2	Precocious puberty	IV	Total	EDP regimen	+	−	+	BWS	3	Mitotane stopped after 2.5 years, stable cerebral metastases. Still on glucocorticoid, mineralcorticoid, and LT4 treatment 6 months after mitotane withdrawal.
2 (F)	2.2	Precocious puberty	IV	Total, spillage	None	+	+	+	−	6	Mitotane stopped after 1 year due to neurological adverse effects, disease free. No PP signs after mitotane withdrawal. Still on glucocorticoid, mineralcorticoid treatment 4 years after mitotane withdrawal. LT4 stopped 3 years after mitotane withdrawal.
3 (M)	5	Precocious puberty	III	Total	EDP + 2° line vincristine + 3° line etoposide/carboplatin	+	+	−	Li Fraumeni	2.3	Dead of disease.
4 (M)	3	Abdominal pain	III	Total	EDP	+	−	+	−	1.5	Mitotane ongoing. Treatment with glucocorticoid and LT4.
5 (F)	4	Precocious puberty	IV	Total	None	+	+	−	−	18	Mitotane stopped after 2.5 years, disease-free, PP treatment with GnRH analogs stopped at age of 11. Glucocorticoid treatment stopped 2 years after mitotane withdrawal.
6 (F)	7.2	Disease detected by ultrasound surveillance due to familiarity	III	Spillage	EDP	+	+	−	Li Fraumeni	1.2	Mitotane ongoing. Treatment with glucocorticoid and fulvestrant.
7 (M)	12	Abdominal pain, loss of weight, headache	IV	Partial	EDP + 2° line gemcitabine/capecitabine + 3° line temozolamide + 4° line pembrolizumab	+	+	+	Li Fraumeni	2.1	Dead of disease.

M—male. F—female. +: present. −: absent. AI—adrenal insufficiency. PP—precocious puberty. CH—central hypothyroidism. BWS—Beckwith–Wiedemann syndrome. EDP—etoposide + doxorubicin + cisplatin. LT4—levothyroxine. GnRH—Gonadotropin Releasing Hormone.

## Data Availability

The data presented in the study are available on request from the corresponding author due to privacy restrictions.

## Abbreviations

The following abbreviations are used in this manuscript:

ACC	Adrenocortical carcinoma
pACC	Pediatric adrenecortical carcinoma
SHBG	Sex hormone binding protein
LH	Luteinizing hormone
FSH	Folllicle stimulating hormone
DHEAS	Dehydroepiandrosterone sulfate
PRL	Prolactin
TSH	Thyroid stimulating hormone
fT4	Free thyroxine
fT3	Free thyronine
ACTH	Adrenocroticotropic hormone
